# Qingfei Paidu decoction for treating COVID-19: A protocol for a meta-analysis and systematic review of randomized controlled trials

**DOI:** 10.1097/MD.0000000000022040

**Published:** 2020-09-04

**Authors:** Yuan Zhang, Hongyan Xie, Yan Li, Tianhao Li, Haipo Yuan, Xiaoxu Fu, Chunguang Xie

**Affiliations:** Hospital of Chengdu University of Traditional Chinese Medicine, Chengdu, Sichuan Province, China.

**Keywords:** COVID-19, meta-analysis, protocol, Qingfei Paidu decoction, systematic review

## Abstract

**Background:**

Coronavirus disease 2019 (COVID-19) is one of the infectious diseases that have seriously threatened global public health since its outbreak in 2019. Due to the complicated Pathogenesis, high infectivity and high fatality rate of COVID-19, there is currently no effective treatment for such epidemic disease. Traditional Chinese medicine has a long clinical history for the prevention and treatment of this kind of acute infectious disease. Qingfei Paidu Decoction (QFPD) is widely used in treating COVID-19 in China. However, there is still a lack of comprehensive and systematic evidence on the effectiveness and safety of Qingfei Paidu Decoction.

**Methods:**

We will search each database from the built-in until May 2020. The English literature mainly searches Cochrane Library, PubMed, EMBASE, and Web of Science, while the Chinese literature comes from CNKI, CBM, VIP, and Wangfang database. Simultaneously we will retrieval clinical registration tests and grey literatures. This study only screen the clinical randomized controlled trials (RCTs) about QFPD for COVID-19 to assess its efficacy and safety. The two researchers worked independently on literature selection, data extraction, and quality assessment. The dichotomous data is represented by relative risk (RR), and the continuous is expressed by mean difference (MD) or standard mean difference (SMD), eventually the data is synthesized using a fixed effect model (FEM) or a random effect model (REM) depending on whether or not heterogeneity exists. Total clinical effective rate, improvement rate of lung CT, adverse events were evaluated as the main outcomes. Effective rate of clinical symptoms, treatment time were secondary outcomes. Finally, meta-analysis was conducted by RevMan software version 5.3.

**Results:**

The results of our research will be published in a peer-reviewed journal.

**Conclusion:**

This systematic review aims to provide new evidence of QFPD for COVID-19 in terms of its efficacy and safety.

**PROSPERO registration number:**

CRD42020200894.

## 1 Introduction

Coronavirus disease 2019 is an acute respiratory infectious disease caused by the new coronavirus type 2 acute respiratory syndrome coronavirus (SARS-CoV-2).^[1]^ COVID-19 patients usually present with respiratory symptoms, such as fever, coughing, sneezing, fatigue, etc. Nearly one-third of patients suffer from at least one coexisting disease.^[2,3]^ Since the first outbreak of the COVID-19 in Wuhan, it has become a key issue that has seriously threatened the public health of people around the world.^[4]^ As this corona virus has the typical characteristics of highly contagious and high fatality rate, until March 2020, the novel coronavirus disease has spread in more than 100 countries around the world.^[5]^ According to reports, a total of 531,630 people worldwide have been infected with the virus, and the death toll is as high as 24,065.^[6]^

There is no effective treatment to control the new coronavirus disease 2019 according to the World Health Organization (WHO) commentary.^[7]^ According to clinical experience, antiviral drugs and symptomatic and supportive treatment are often used. However, its therapeutic effect still needs further evaluation.^[8,9]^ Therefore, it is imperative that we need to seek new treatment methods and measures to prevent the progression and prevalence of the disease.^[10]^

However, traditional Chinese medicine and Chinese herbal medicine have accumulated rich clinical experience and effective formulas in the prevention and treatment of epidemics, such as SARS in 2003. In this epidemic, Both of them also played a huge role in the prevention and control of the new coronavirus 2019.^[11,12]^ After the outbreak of the coronavirus disease, the National Health Commission of the People's Republic of China formulated and issued the“Diagnosis and Treatment Protocol for Novel Coronavirus Pneumonia (Trial Version),” which is based on the clinical manifestations of the disease, pathology, and accumulated experience in the diagnosis and treatment.^[13]^ Qingfei Paidu Decoction (QFPD) appears in this plan as one of the recommended Chinese medicine prescriptions. Qingfei Paidu Decoction is a traditional Chinese medicine compound first created by Zhang Zhongjing. It is composed of *Ma Xing Shi Gan Decoction, She Gan Ma Huang Decoction, Xiao Chai Hu Decoction,* and *Wu Ling San*. *Herba Ephedrae, Radix Glycyrrhizae, Semen Armeniacae Amarum, Gypsum Fibrosum, Ramulus Cinnamomi, Rhizoma Alismatis, Polyporus Umbellatus, Rhizoma Atractylodis Macrocephalae, Poria, Radix Bupleuri, Radix Scutellariae, Rhizome Pinelliae Preparata, Rhizoma Zingiberis Recens, Radix Asteris, Flos Farfarae, Rhizoma Belamcandae, Herba Asari, Rhizoma Dioscoreae, Fructus Aurantii Immaturus, Pericarpium Citri Reticulatae, Herba Pogostemonis* make up the Qingfei Paidu Decoction. In terms of modern pharmacological analysis, the prescription has multiple functions of antiviral, anti-inflammatory, immune regulation, and antipyretic. It has also been clinically proven to have a good effect on COVID-19.^[14]^ Therefore, we intend to collect randomized controlled trials (RCTs) about QFPD for COVID-19 based on the basis of evidence-based medicine, and conduct a meta-analysis of its efficacy and safety to provide higher quality clinical evidence for Chinese medicine treatment of COVID-19.

## 2 Methods

### 2.1 Protocol registration

The systematic review protocol has been registered on the prospero website as CRD42020200894 (https://www.crd.york.ac.uk/prospero/#recordDetails). It is reported following the guidelines of Cochrane Handbook for Systematic Reviews of Interventions and the Preferred Reporting Items for Systematic Reviews and Meta-analysis Protocol (PRISM).^[15]^ We will update our protocol for any changes in the entire research process if needed.

### 2.2 Inclusion criteria

#### 2.2.1 Study design

The study only select clinical randomized controlled trials of QFPD for COVID-19 published in both Chinese and English. However, animal experiments, reviews, case reports, and non-randomized controlled trials are excluded.

#### 2.2.2 Participants

This study included patients who had been clearly diagnosed with the new coronavirus disease. Except that participants must be over 18 years old, there were no strict restrictions on gender and severity of the disease.

#### 2.2.3 Intervention

The test group uses QFPD. The control group can be treated with other treatments except QFPD. There are no obvious restrictions on the dosage of therapeutic drugs and specific intervention routes.

#### 2.2.4 Outcomes

The primary outcomes include total clinical effective rate, improvement rate of lung CT, adverse events. Secondary outcomes are mainly composed of effective rate of clinical symptoms treatment time.

### 2.3 Search methods

#### 2.3.1 Electronic searches

We will retrieve each database from the built-in until May 2021. The English literature mainly searches Cochrane Library, PubMed, EMBASE, and Web of Science. While the Chinese literature comes from CNKI, CBM, VIP, and Wangfang database. We adopt the combination of heading terms and free words as search strategy which decided by all the reviewers. Search terms: Qingfei Paidu Tang, Qingfei Paidu Decoction, 2019 novel coronavirus disease, COVID-19, coronavirus disease 2019, 2019 novel coronavirus infection, 2019-nCoV disease. We will simply present the search process of the Cochrane library (Table 1). Adjusting different search methods according to different Chinese and English databases.

**Table 1:**
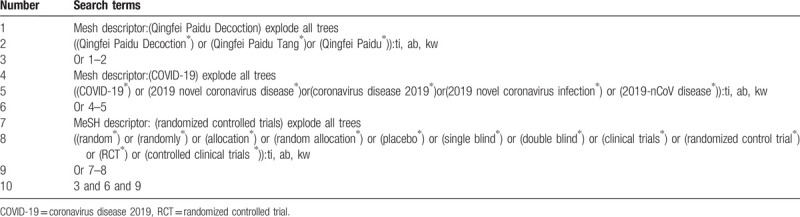
Cochrane library search strategy.

#### 2.3.2 Searching other resources

At the same time, we will retrieve other resources to complete the deficiencies of the electronic databases, mainly searching for the clinical trial registries and grey literature about QFPD for COVID-19 on the corresponding website.

### 2.4 Data collection and analysis

#### 2.4.1 Selection of studies

**Figure 1:**
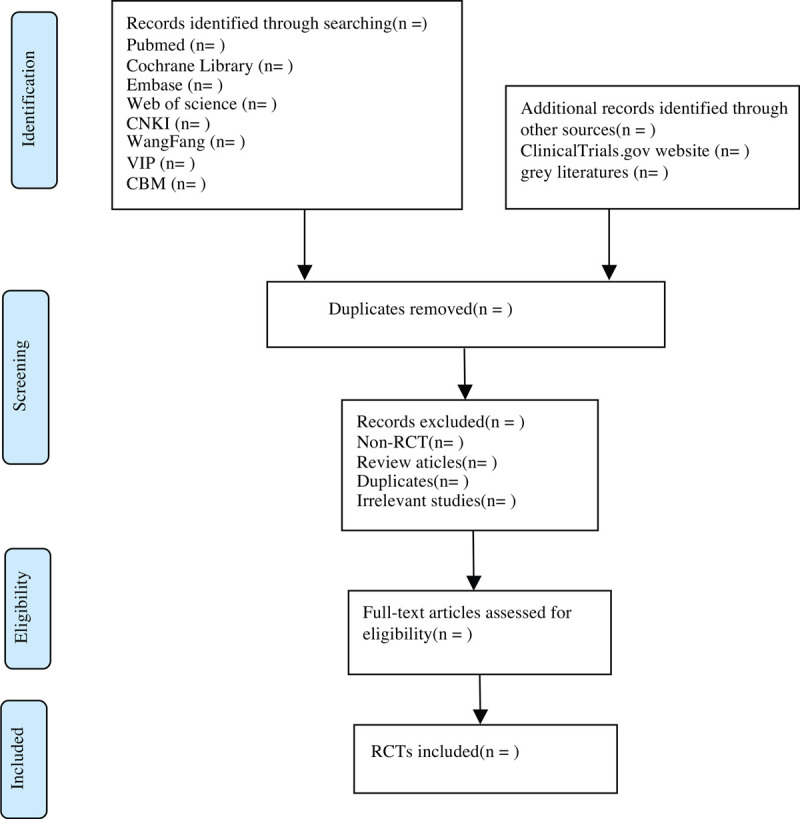
Flow chart of the study selection.

Import all literatures that meet the requirements into Endnote X8 software. First of all, two independent reviewers initially screened the literatures that did not meet the pre-established standards of the study by reading the title and abstract. Second, download the remaining literatures and read the full text carefully to further decide whether to include or not. Finally, the results were cross-checked repeatedly by reviewers. If there is a disagreement in the above process, we can reach an agreement by discussing between both reviewers or seek a third party's opinion. Flow chart of the study selection (Fig. 1) will be used to show the screening process of the study.

#### 2.4.2 Data extraction and management

According to the characteristics of the study, we prepare an excel form for data collection before data extraction. Outcome indicators for eligible studies were independently extracted and filled in the data extraction form by two reviewers. The main data extracted are as follows: title, author, year, fund source, sample size, age, sex, duration of disease, interventions, outcome measures, adverse reactions, etc. If there are something unclear, you can not hesitate to contact authors of more detailed information. The above information was finally cross-checked by two reviewers.

#### 2.4.3 Assessment of risk of bias in included studies

The quality assessment of RCTs adopts the risk of bias (ROB) assessment tool provided by the Cochrane Handbook. The following seven items, such as random sequence generation, allocation concealment, blinding of participants and personnel, blinding of outcome assessment, incomplete outcome data, selective outcome reporting, and other bias, are evaluated by three grades of “low bias,” “high bias,” and “unclear bias.” The discrepancies will get a consistent conclusion by discussing between both reviewers or seeking the third-party consultation.

#### 2.4.4 Data analysis

Different evaluation methods are selected according to the different efficacy indicators. For the dichotomous data, we will choose the effect scale indicator relative risk (RR) with 95% confidence interval (CI) to represent. While the continuous data is expressed as mean difference (MD) or standardized mean difference (SMD) with 95% CI depending on whether the measurement scale is consistent or not. Review Manager software version 5.3 provided by the Cochrane Collaboration will be performed for data synthesis and analysis. The dichotomous data is represented by RR, continuous data is expressed by MD or SMD. If there is no heterogeneity (*I*^2^ < 50%, *P* > 0.1), the data are synthesized using a fixed effect model. Otherwise (*I*^2^ ≥ 50%, *P* < 0.1), a random effect model is used to analyze. Then subgroup analysis will be conducted basing on the different causes of heterogeneity. If a meta-analysis cannot be performed, it will be replaced by a general descriptive analysis.

## 3 Discussion

Due to the high lethality and epidemic of the new coronavirus disease, it has attracted the attention of various countries around the world.^[16–17]^ The current clinical treatment is mainly based on antiviral drugs and symptomatic support, and no unified treatment method has been established.^[18]^ Therefore, this pushes us to explore and research novel drugs to fill the current treatment deficiencies.

Traditional Chinese medicine and Chinese herbal medicine have a long history of treating infectious diseases such as malaria in ancient China. It not only has obvious advantages in effective treatment, but also has fewer side effects. During the prevalence of the coronavirus, the clinical efficacy data of the traditional Chinese medicine compound QFPD showed that it has the effect of reducing patient mortality and improving respiratory symptoms. Therefore, this study intends to comprehensively evaluate the safety and safety of the efficacy of QFPD in COVID-19 patients through systematic reviews and meta-analysis. In other words, it can also provide new therapy for early control of the virus and defeat epidemic.

## Author contributions

**Conceptualization:** Yuan Zhang, Hongyan Xie.

**Data curation:** Yan Li, Tianhao Li.

**Funding acquisition:** Chunguang Xie.

**Methodology:** Yuan Zhang, Yan Li.

**Project administration:** Chunguang Xie.

**Software:** Haipo Yuan, Hongyan Xie.

**Supervision:** Xiaoxu Fu.

**Writing – original draft:** Yuan Zhang.

**Writing – review & editing:** Chunguang Xie.
